# Stercoral Colitis as a Cause of Altered Mental Status in a Patient With Cerebral Palsy

**DOI:** 10.7759/cureus.33040

**Published:** 2022-12-28

**Authors:** Rishi Chowdhary, Benjamin Liu, Muhammad Husnain

**Affiliations:** 1 Department of Internal Medicine, Shri Dharmasthala Manjunatheshwara College of Medical Sciences, Dharwad, IND; 2 Department of Internal Medicine, MetroHealth Medical Center, Cleveland, USA

**Keywords:** enema treatment, altered mental status, fecal disimpaction, elderly patients, constipation, cerebral palsy (cp)

## Abstract

Stercoral colitis (SC) is a rare inflammatory colitis that occurs due to increased intraluminal pressure from impacted fecal content in the colon. Chronic constipation is the major risk factor for this condition. Delayed diagnosis is associated with high morbidity and mortality, with complications ranging from colonic perforation to intestinal ulcers. Patients usually present with non-specific symptoms, with advanced cases presenting with acute abdomen pain. This condition can be fatal if not recognized early and promptly treated. Early detection can often be difficult in elderly patients with dementia, stroke, or other neurologic disorders that cause altered mental status (AMS). Therefore, AMS in patients with severe constipation should be a substantial reason to consider stercoral colitis as a differential diagnosis. Here, we describe a case of stercoral colitis in a 59-year-old woman with non-verbal cerebral palsy who had acute metabolic encephalopathy from her stercoral colitis and was successfully treated with colonoscopic fecal disimpaction and an aggressive bowel regimen.

## Introduction

Stercoral colitis (SC) is a rare form of inflammatory colitis that is caused by increased intraluminal pressure from impacted fecal matter in the colonic segments [1]. Involvement of the sigmoid colon or rectum accounts for the vast majority of instances of SC [2]. SC can manifest in various ways, ranging from circumferential inflammation around an impacted fecaloma to colonic ulceration and perforation. SC is associated with a high morbidity and mortality rate, with a mortality rate of 32-60% if colonic perforation occurs [1]. In 27% of patients with stercoral colitis, there are intestinal ulcers, of which 77% of these ulcerations happen in the rectosigmoid or sigmoid colon [3].

Advanced age, dementia, chronic concomitant illness, cancer, and non-ambulatory status are all risk factors for stercoral colitis, with persistent constipation of any kind being the predominant predisposing condition [4]. Chronic neurologic and psychiatric illnesses may delay the diagnosis of SC because they hinder thorough history-taking and conceal physical examination findings [5].

When there is only a simple fecaloid impaction, fecal impaction may not be symptomatic, but it can cause complications, including gut wall inflammation and infection, ulcer development, hemorrhoidal thrombosis, ischemia pressure necrosis, colonic perforation, peritonitis, and septic shock [6]. Most patients have an abdominal exam suggestive of an acute abdomen when they enter the emergency room (ER). Other non-specific symptoms may include abdominal distension, absence of gas-feces discharge, nausea and vomiting, and anorexia. Fever, chills, lower gastrointestinal bleeding, and hypotension are some of the symptoms that can appear when complications occur [6].

Since the first recorded case in 1894, there have only been approximately 200 cases of SC reported [1]. This is a complication of what initially appeared as simple constipation. Early identification and treatment of stercoral colitis are essential to reduce overall morbidity and mortality [4]. The incidence of SC is predicted to rise with the aging of our population; thus, clinicians need to be aware of this illness, key diagnostic tools, and treatment options [4]. 

Here, we describe a case of stercoral colitis in a 59-year-old woman with non-verbal cerebral palsy who had acute metabolic encephalopathy from her stercoral colitis and was successfully treated with colonoscopic fecal disimpaction and an aggressive bowel regimen.

## Case presentation

A 59-year-old woman with a past history of non-verbal cerebral palsy, chronic idiopathic constipation, positive fecal immunochemical test (FIT) (pending an outpatient colonoscopy), and mitral valve prolapse presented to the emergency room with a chief concern of altered mental status. The patient was chronically on valproic acid and quetiapine for her cerebral palsy and had previously been treated with multiple aggressive bowel regimens for her chronic constipation. On admission, the patient’s caregiver’s expressed concern about the loss of appetite and decreased oral intake over the past three days, with her last bowel movement being two days back. Given the patient’s non-verbal status, the patient’s baseline status was difficult to elicit, and we had to rely on the caregiver's observations. Nevertheless, the caregiver stated that the patient was less interactive than usual. Vitals showed a blood pressure of 135/117, a heart rate of 95, a temperature of 36.9, a respiratory rate of 22, and a percent oxygen saturation of 95%.

On physical examination, the patient was somnolent, lying in bed, not in acute distress, and was attentive to persons in the room despite her non-verbal status. A complete neurological exam was impossible as the patient was not cooperative; however, there were no gross deficits on her cranial nerve exam and no focal deficits noted in her extremities. There was a good capillary refill and 2+ distal pulses. The abdomen appeared tense and distended; however, the patient did not react to superficial palpation but only to deep palpation. Bowel sounds were present. There was also noticeable distension in the suprapubic area that did not appear to be tender either. 

Routine altered mental status workup was unremarkable, including head imaging, thyroid stimulating hormone levels, vitamin B12, respiratory panel, syphilis, and urinalysis. Table 1 shows the relevant labs that were done on admission with the reported normal ranges. 

**Table 1:** Measured lab values with reference ranges.

Parameters	Measured values	Reported ranges
Platelet count	73,000/mm^3^	150,000-400,000/mm^3^
Serum creatinine	0.27 mg/dL	0.6-1.2 mg/dL
Blood urea nitrogen	12 mg/dL	7-18 mg/dL
Serum albumin	2.8 g/dL	3.5-5.5 g/dL

**Figure 1:**
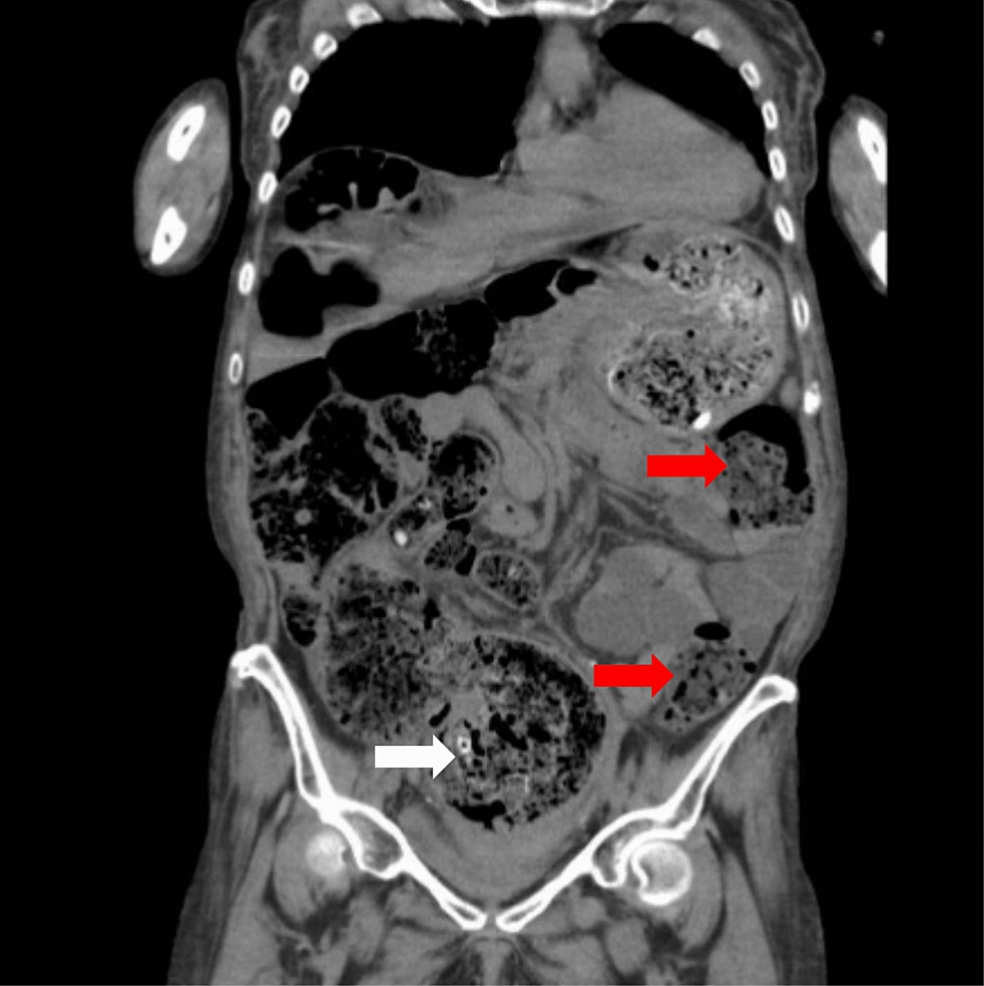
Overall stool burden (red arrows) with a preliminary view of the stoolball (white arrow). Red arrows display the overall stool burden. White arrow displays the preliminary view of the stoolball.

Computed tomography of the abdomen and pelvis was performed, demonstrating marked stool in the colon with distention of the rectum up to 10.1 cm (Figure 1).

**Figure 2:**
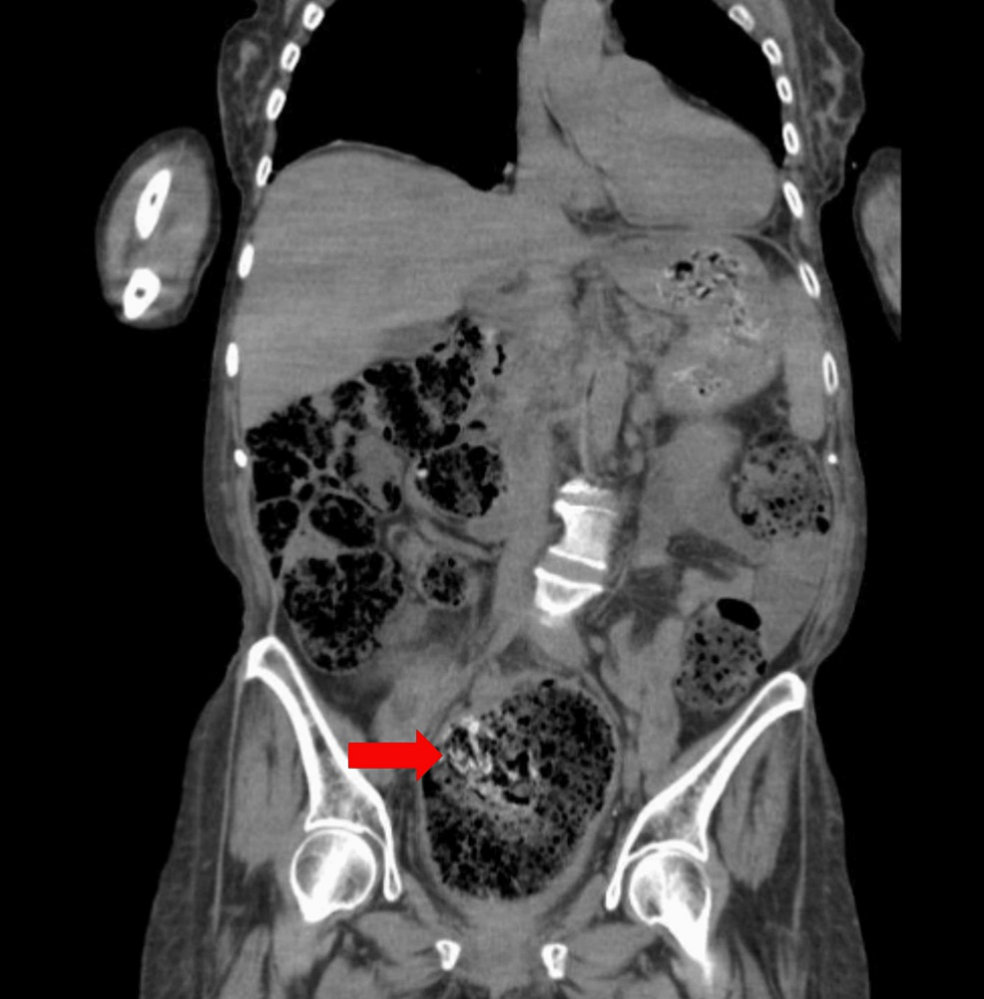
Dramatic view of the rectosigmoid stoolball (red arrow). Red arrow shows the rectosigmoid stoolball with mild circumferential thickening.

Additionally, a mild circumferential wall thickening of the rectosigmoid colon is seen (Figure 2), suggestive of non-perforated fecal impaction with SC.

Oral MiraLAX and senna were started, following which the patient had extremely loose Bristol stool scale 7 bowel movements. The patient had not passed urine for over 14 h and had retention on the bladder scan, resulting in Foley catheter (Bardex, West Sussex, UK) placement. Her quetiapine was held. After two days of consecutive treatment, the patient’s mental status improved closer to the baseline per caregiver; however, her abdominal exam was only mildly improved. Her ongoing bowel movements continued to be loose and watery. Therefore, a bedside manual disimpaction was attempted. Unfortunately, this was unsuccessful, although a very hard stoolball was palpated in the rectum. Serial abdominal exams showed no signs of peritonitis, though abdominal x-rays continued to show significant stool burden. Her acute encephalopathy was suspected to be metabolic, secondary to her severe constipation with SC. As a result, more aggressive management of her constipation was needed, and gastroenterology was consulted. Given the stoolball, her prior positive FIT test, and her pending outpatient colonoscopy, a decision was made to do an inpatient colonoscopic disimpaction. In anticipation, daily tap water enemas and 8L of Golytely (Novel Laboratories, New Jersey, USA) were given via nasogastric tube.

Despite being a challenging procedure in the presence of an atonic and looping colon, a colonoscopy was completed with 95% of the colon visible. Colonoscopy also demonstrated an abnormal stoolball in the rectum that was unfortunately not suctionable. Other than melanosis coli of the cecum, there were no abnormal findings. SC was felt to be the cause of the positive FIT. After the colonoscopy, the patient was continued on a rigorous bowel regimen of linaclotide, MiraLAX, senna, milk of magnesia, and Dulcolax as needed, which led to improvement in the abdominal exam. Diet was advanced with the return to normal Bristol stool 4 bowel movements, and the patient was discharged in stable condition.

## Discussion

SC is an unusual and sometimes fatal side effect of chronic constipation. The significant morbidity of stercoral colitis makes it crucial to identify risk factors to ensure prompt diagnosis and treatment. It is believed that severe chronic constipation, which is present in 60-81% of patients, is the primary cause causing the development of SC [7]. Chronic constipation is a frequent geriatric issue; thus, the median age of onset for stercoral colitis is 62, and children are rarely afflicted by it [8]. Women are also more likely than men to develop constipation [9].

According to numerous large-scale studies, 15-20% of people over 60, 20-37% of those over 84, and up to 80% of people who need long-term care (in nursing facilities) have chronic constipation [10,11]. This is related to several risk factors, such as tooth loss or poorly fitted dentures, poor dietary habits, insufficient hydration intake, a decline in physical activity, and an increase in the number of medications older patients take each day [9]. Additionally, many drugs, including opiates, tricyclic antidepressants, non-steroidal anti-inflammatory drugs (NSAIDs), steroids, aluminum-based antacids, antipsychotics, verapamil, and immunosuppressants, may cause constipation [12,13]. In our case, the patient was a 59-year-old woman with non-verbal cerebral palsy and chronic constipation who was on regular medications like valproate and quetiapine.

Severe, chronic constipation or fecal impaction results in the formation of a hard fecaloma that reduces transmural circulation, potentially causing tissue ischemia and necrosis, accompanied by the development of a necrotic stercoral ulcer that may perforate if not adequately treated. The anterior rectum close to the peritoneal reflection, the antimesenteric border of the rectosigmoid junction, and the apex of the sigmoid colon are the areas most prone to stercoral colitis due to the decreasing water content of stool in the distal intestinal tract, relatively narrow diameter, and poor tissue perfusion [14]. The blood supply to these regions relies on an anastomosis between branches of the inferior mesenteric and superior rectal arteries at a site referred to as Sudeck's point, which is often inadequate or absent [1].

The symptoms of stercoral colitis can be asymptomatic or ill-defined; there is no single defining symptom for this condition. Patients may have only abdominal pain at the initial stages. Appendicitis, gastrointestinal hemorrhage, and diverticulitis are a few of the abdominal conditions that stercoral colitis may imitate [7,15]. Cognitive disabilities such as cerebral palsy, immobility, rectal hyposensitivity, inadequate hydration, and the use of constipating drugs are risk factors for chronic constipation and SC [16,17]. SC should therefore be included in the differential diagnosis of patients presenting to the emergency department with abdominal pain or rectal bleeding when the following conditions are present: patient age is >65, history of or risk factors for chronic constipation, living in a nursing home/long-term care facility, previous neurological or mobility issues, and chronic opioid usage.

In our case, the patient was non-verbal and could not elicit any abdominal pain. Therefore, SC's primary suspects were altered mental status and abdominal distension with chronic constipation. Due to the variability in clinical presentation, clinicians should have a low threshold when ordering diagnostic imaging to confirm SC and rule out perforation in these individuals. Rarely, as in our instance, SC can worsen due to urine retention from the fecaloma's compressive influence on the bladder.

With 82-90% accuracy, computerized abdominal tomography (CT) is the most sensitive modality for detecting perforations, intraperitoneal air, and extraluminal fecal contents [18]. IV contrast is recommended if the patient's renal function is adequate. Typically, a CT scan will show the following things: (1) focal thickening of the colonic wall, which could indicate edema from ischemia and ulceration; (2) pericolonic fat stranding; and (3) extraluminal gas or an abscess if a perforation occurs [14].

Furthermore, the prognostic usefulness of CT results is substantial, with findings of ascites, dense mucosa, perfusion defects, or anomalous gases more frequently associated with increased mortality [19]. Therefore, CT is considered statistically valuable in predicting fatal from non-fatal SC [19]. Only 30% of colonic perforations show open air under the diaphragm on abdominal and chest x-rays [18], suggesting that x-rays have poor diagnostic value and do not rule out complications in the ED should the clinical suspicion be high. Leukocytosis, lactic acidosis, and increased inflammatory markers are a few examples of laboratory abnormalities [16]. However, they do not reveal the underlying illness as they are not specific. In our case, the CT abdomen/pelvis demonstrated marked stool in the colon with rectal distention and mild circumferential wall thickening of the rectosigmoid colon with no extraluminal gas, suggesting non-perforated fecal impaction with SC.

To prevent late-stage complications, treatment includes timely fecal disimpaction and an intensive bowel regimen [3]. Rectal enemas and oral medications, such as senna, magnesium citrate, MiraLAX, and Dulcolax, are frequently used in bowel regimens. Due to the significant pressure a fecaloma mass places on the colonic walls, manual disimpaction is usually successful at lowering intraluminal pressure inside the colon and the risk of ulceration. If one cannot relieve the blockage manually and the patient has no signs of peritonitis or impending perforation, endoscopically guided disimpaction should be attempted. If CT indicates a defect in the colonic wall, manual disimpaction should not be attempted. Intestinal regimens can treat 52% of patients without surgery, according to research by Gau et al. [1]. Open laparotomy, extensive peritoneal lavage, Hartmann's operation with a colostomy, or segmental resection with primary anastomosis and diverting colostomy are all surgical options for treating stercoral perforation [1]. During the Hartmann procedure, a segment of the affected sigmoid colon is resected, an end colostomy is created, and the distal rectal stump is closed by stapled sutures or sewn by hand. The resulting Hartmann pouch ultimately becomes a blind segment of the colon that extends from the anus to the sealed stump [20]. 

According to the Ünal et al. study, the factor most closely associated with mortality was the length of the affected colon segment, with 57.1% of patients with colon involvement measuring >40 cm dying [5]. As in our case, after starting the oral bowel regimen, manual disimpaction was tried but was unsuccessful, following which gastroenterology was consulted for inpatient colonoscopic disimpaction. As discussed in the Marget et al. report, our patient was put on maintenance laxative therapy before discharge to reduce the risk of recurrence [13].

## Conclusions

Stercoral colitis presents with relatively non-specific signs but should be a consideration in at-risk individuals to prevent potentially fatal complications. Early diagnosis and management are critical to lowering the risk of complications and improving mortality. Patients who are unable to provide a history or cooperate for a physical exam are incredibly challenging, but these patients tend to be more at risk for this disease. 
